# ddPCR for the Detection and Absolute Quantification of Oropouche Virus

**DOI:** 10.3390/v16091426

**Published:** 2024-09-07

**Authors:** Elena Pomari, Andrea Matucci, Silvia Accordini, Rebeca Passarelli Mantovani, Natasha Gianesini, Antonio Mori, Concetta Castilletti

**Affiliations:** Department of Infectious, Tropical Diseases and Microbiology, IRCCS Sacro Cuore Don Calabria Hospital, 37024 Negrar di Valpolicella, Verona, Italy; andrea.matucci@sacrocuore.it (A.M.); silvia.accordini@sacrocuore.it (S.A.); rebeca.passarellimantovani@sacrocuore.it (R.P.M.); antonio.mori@sacrocuore.it (A.M.); concetta.castilletti@sacrocuore.it (C.C.)

**Keywords:** ddPCR, quantification, RNA, Oropouche virus

## Abstract

Background: Oropouche virus (OROV) is a segmented RNA virus belonging to the genus *Orthobunyavirus* in the family *Peribunyaviridae*. Herein, an in-house droplet digital PCR (ddPCR) assay was used for the detection and quantification of OROV. Methods: The ddPCR reaction was assessed as duplex assay using the human housekeeping gene *RPP30*. Limit of detection (LoD) analysis was performed in whole blood, serum, and urine. The assay was executed on a total of 28 clinical samples (whole blood n = 9, serum n = 11, and urine n = 8), of which 16 specimens were tested positive at the routine molecular diagnostics (endpoint and real-time PCRs). Results: The LoD of the ddPCR performed using 10-fold serial dilution of OROV detected up to 1 cp/µL in all the biological matrices. Compared to the routine molecular diagnostics, the ddPCR assay showed 100% sensitivity for whole blood and serum and 75% for urine, highlighting higher positive rate of ddPCR. Conclusion: We have established a quantitative RNA detection method of OROV with high sensitivity and specificity based on ddPCR. This test is capable of quantitatively monitoring the viral load of OROV and can contribute, in addition to laboratory diagnosis, to shed light on the pathogenesis, filling in the knowledge gaps of this neglected disease and to the vector control programs.

## 1. Introduction

The Oropouche virus (OROV) is an important arthropod-borne virus in the *Peribunyaviridae* family that can cause febrile illnesses, and it is widely distributed in tropical regions such as Central and South America [1]. The Oropouche virus is spread to people primarily by the bite of an infected biting midge (*Culicoides paraensis*) in an urban cycle [1]. In May 2024, the Ministry of Public Health of Cuba reported the first ever outbreak of OROV disease [2]. Due to recent, very worrisome changes in the observed clinical and epidemiological characteristics of Oropouche fever, the Pan American Health Organization (PAHO) has raised the risk level for the region to high [3]. PAHO has highlighted the need to strengthen epidemiological and entomological surveillance and reinforce preventive measures in the population. It is critical to expand the scarce knowledge of this neglected disease, including new possible routes of transmission and new vectors that could affect both the general population and vulnerable groups, such as pregnant women. As of 30 July 2024, 8078 confirmed cases of OROV have been reported in the following five countries in the region of the Americas: the Plurinational State of Bolivia (n = 356), Brazil (n = 7284), Colombia (n = 74), Cuba (n = 74), and Peru (n = 290) [3]. The prevalence of OROV infections is likely underestimated in the Americas, where more than ten million cases of dengue were reported between epidemiological weeks 1 and 26 of 2024 [4,5]. In late May–early June 2024, OROV fever cases in two travelers from Cuba were notified as the first imported cases of OROV disease from our hospital in Italy [6], suggesting the importance of expanding the travel history for considering a diagnosis of OROV fever in returning travelers from Central or South America, including Cuba and other Caribbean countries too. As of 9 August 2024, between June and July 2024, 19 imported cases of OROV disease have been reported in Spain (n = 12), Italy (n = 5), and Germany (n = 2) [7]. It is noteworthy that PAHO has indicated, as diagnostic sampling, only serum and eventually cerebrospinal fluid (CSF) for cases presenting with aseptic meningitis (a rare complication of Oropouche fever) [8]. Based on our recent findings in the diagnosis of OROV infection in the EU, we also suggest the use of whole blood and urine to increase the possibility of a definite diagnosis [9]. OROV is a segmented RNA virus and, nowadays, the detection of viral RNA by a PCR-based molecular method (conventional RT-PCR and real-time RT-PCR) is the most frequently applied laboratory test due to its higher sensitivity and specificity. So far, no commercial PCR-based diagnostic tests have been developed in order to monitor and diagnose OROV, in-house OROV developed tests target a conserved 5′ region of the *S* segment [10,11], and one in particular has been recommended by PAHO [12]. In recent decades, a new generation of PCR (i.e., droplet digital PCR, ddPCR), has been introduced into the field of molecular virology for detection and, above all, the quantification of viruses [13,14]. Compared to the more commonly used real-time PCR (rt-PCR), ddPCR provides absolute quantification of nucleic acid copies in samples without the need for standard curves, and it also has the ability to detect lower genomic yield. The ddPCR is based on microfluidics technology, which allows for the generation of multiple reaction partitions that work as individual reactions. Based on the positive or negative fraction and following the Poisson distribution, it is possible to determine the absolute concentration of the target of interest in terms of the number of copies per microliter in the ddPCR reaction. In this study, we used the PAHO primers/probe set [12] to set up a ddPCR and to assess its analytical and clinical performance for detecting and quantifying genomic RNA of OROV on whole blood, serum, and urine matrices and clinical samples.

## 2. Materials and Methods

### 2.1. Cell Line and Virus

A viral isolate (named OROV-IRCCS-SCDC_1/2024) was obtained from the serum sample of a patient returning from Cuba on May 2024, with laboratory-confirmed OROV, in the IRCCS Sacro Cuore Don Calabria (SCDC) Hospital BLS3 (biosafety level 3) laboratories. Briefly, the serum sample was diluted 1:10 in minimum essential medium (MEM, Gibco, Thermo Fisher Scientific, Waltham, MA, USA) and then inoculated onto a semiconfluent monolayer of Vero E6 cells (ATCC CRL-1586) grown with MEM containing 1% penicillin-streptomycin (Gibco), 1% L-Glutamine (Gibco, Thermo Fisher Scientific, Waltham, MA, USA), and 2% fetal bovine serum (Gibco, Thermo Fisher Scientific, Waltham, MA, USA). Cytopathic effect (CPE) appearance was observed by light microscope. The culture medium was harvested, low-speed centrifuged, and the supernatant was aliquoted and stored at −80 °C.

### 2.2. Viral Titration

A day prior to the infection, Vero E6 cells were grown to semiconfluence with 10% FBS MEM in a 96-well tissue culture plate at 37 °C, 5% CO_2_.

On the day of infection, a series of 3-fold dilutions of the original virus sample were prepared in MEM without FBS. Each dilution was tested in four replicates. Once the dilution plate was prepared, the 10% FBS MEM medium was removed from the monolayers, and 100 µL of each dilution was added onto the cells. The plate was kept at 37 °C, 5% CO_2_.

In every plate, four wells were used as the no-virus control. The plate was incubated and observed daily to monitor the development of a cytopathic effect (CPE) by the light microscope. CPE was evaluated and viral titer was calculated using Reed and Muench’s [15] calculation and expressed as tissue culture infectious dose (TCID_50_)/mL.

### 2.3. Clinical Samples and in House PCRs

A total of 28 clinical samples (EDTA whole blood n = 9, serum n = 11, and urine n = 8) collected at diagnosis or during follow-up were previously screened in our laboratory with routine real-time PCR diagnostic tests specific for imported Flavivirus fevers (including Dengue, Zika, Chikungunya) and then for OROV, based on in-house endpoint PCR using Lambert primers [11] and the OROV real-time PCR (PAHO rtPCR) [12], with both targeting the *S* segment. Tests were performed using BioRad (Hercules, CA, USA) reverse transcriptase mixes (iScript Reverse Transcription Supermix for RT-qPCR or Reliance One-Step Multiplex RT-qPCR Supermix). Sanger sequencing was performed using the same amplification primers on positive endpoint samples to confirm OROV infection. Among the total of samples, n = 16 were tested positive and n = 12 negative to OROV. All samples were negative for Flaviviruses.

### 2.4. RNA Extraction

RNA was isolated from the whole blood and urine using EZ1 Advanced XL System with the EZ1 DSP Virus Kit (Qiagen, Hilden, Germany); both matrixes were pre-diluted 1:4 (V:V) with PBS and then mixed with ATL buffer following the manufacturer’s instructions and eluted in 60 μL. RNA from serum was instead isolated using the QIAamp Viral RNA Mini kit (Qiagen, Hilden, Germany) from 140 μL and eluted in 60 μL. The isolated RNA was stored at −80 °C until use by ddPCR.

### 2.5. ddPCR

The ddPCR procedure was performed with primers/FAM TAQman probe set targeting a tract of *S* segment of OROV genome used in PAHO-rtPCR [12]. In addition, the HEX probe assay for the human Ribonuclease P protein subunit p30 (*RPP30*) (ddPCR CNV Assay Validated, BioRad, Hercules, CA, USA) was used as the human housekeeping gene. The duplex PCR was performed using the One-step RT-ddPCR Advanced Kit for Probes (BioRad, Hercules, CA, USA) and the reaction mixture was assembled as follows: supermix 5.5 μL, reverse transcriptase 20 U/µL 2.2 μL, DTT 15 mM 1.1 μL, OROV primers at 900 nM, and probe at 250 nM as the final concentration, 20× *RPP30* Assay 2 μL and RNA template 7 μL, in a final volume of 22 μL. The QX200 droplet generator (Bio-Rad, Hercules, CA, USA) was used to convert 20 μL of each reaction mix into droplets. The droplet-partitioned samples were transferred to a 96-well plate, sealed and processed in a C1000 touch Thermal Cycler (Bio-Rad) under the following cycling protocol: 25 °C hold for 3 min, 50 °C for 60 min reverse transcription, 95 °C for 10 min for enzyme activation, 95 °C for 30 s for denaturation, 60 °C for 60 s for annealing/extension for 50 cycles, 98 °C 10 min for enzyme deactivation, followed by 30 min at a 4 °C hold. The amplified samples were then transferred and read in the FAM (OROV) and HEX (*RPP30*) channels using the QX200 reader (BioRad, Hercules, CA, USA). The experiments were performed using a negative control (negative biological matrix), the no-template control (water) and a positive control (OROV strain isolate IRCCS-SCDC_1/2024). Discordant samples were repeated in duplicate (then merged for quantification). Data were analyzed using the QXManager 1.2 Standard Edition Software (Bio-Rad, Hercules, CA, USA) and expressed as copies/µL (cp/µL) in ddPCR reaction.

### 2.6. Analytical Sensitivity (Limit of Detection, LoD) Analysis

The LoD calculation of ddPCR was performed using ten-fold serial dilutions of OROV RNA extracted from the viral strain IRCCS-SCDC_1/2024 (stock titer 10^5^ TCID_50_/mL) ranging from 10^4^ to 10^−2^ TCID_50_/mL generated in nuclease-free water. Regarding whole blood and urine (both diluted 1:4 with PBS, V:V before extraction) [16], and serum, the serial dilutions were generated by spiking the OROV viral strain 10^5^ TCID_50_/mL in each biological matrix (from healthy donors). An additional point was tested as negative control (no RNA or viral strain added) for both water and biological matrices. For the biological matrices, the RNA was then extracted as described above. The singleplex assay for OROV was performed in duplicate on water and the duplex OROV/*RPP30* assay in triplicate on biological matrices. LoD was calculated as the last dilution with all replicates detected OROV positive. Standard deviation (SD) and coefficient of variation (CV%) were calculated. For each analysis, the results were expressed as copies/µL (cp/µL).

### 2.7. Statistics

Sensitivity and specificity for the clinical evaluation were analyzed by a two-way contingency table using a diagnostic test evaluation calculator (https://www.medcalc.org/calc/diagnostic_test.php, accessed on 12 July 2024). Correlation and regression analyses and graphs were performed using Jamovi software version 2.5.3.0. The Shapiro–Wilk test was used to test the normal distribution and Pearson’s correlation coefficient was used for the correlation analysis.

## 3. Results

### 3.1. Limit of Detection Analysis (LoD)

**Figure 1:**
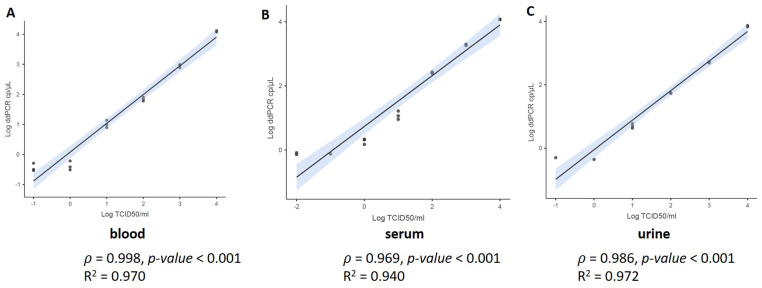
Evaluation of the ddPCR assay LoD performance. Standard curves were obtained from 10-fold serially diluted samples of the reference viral strain (10^5^ TCID_50_/mL) in three biological matrices (whole blood (**A**), serum (**B**) and urine (**C**)). The curves include also the negative point (no viral strain). Pearson statistics was used.

The analytical sensitivity of the assay was assessed by determining the LoD as the lowest viral titer expressed in cp/µL that can be detected by ddPCR. The LoD of the ddPCR performed on 10-fold serial dilution of OROV RNA in water detected up to 1 cp/µL (in Supplementary Materials, Table S1). No OROV target was reported in the diluent control. Then, we performed the LoD analysis in different matrices by ddPCR spiking the viral strain in the whole blood, serum, and urine. We assessed the duplex assay targeting OROV and *RPP30*. The assay was performed in triplicate. The viral copies were detected up to 1 cp/µL in all replicates for all samples (data are reported in Supplementary Material Table S2). In particular, we observed a strong correlation between quantification (cp/μL) and viral titer (*p* > 0.98, *p*-value < 0.001) with a regression coefficient R^2^ >0.94 (Figure 1) for all the biological matrices. Concerning the variability of data, OROV and *RPP30* results were equally repeatable, with CV generally ≤30% for all matrices.

### 3.2. Clinical Samples

We investigated a total of 28 clinical samples with positive (n = 16) or negative (n = 12) results at the routine diagnosis for OROV with rt-PCR. Table 1 summarizes the ddPCR results for each biological matrix (whole blood, serum, and urine). Positive detection was determined even if there were <3 droplets. Supplementary material reports the detailed data (Supplementary Material, Table S3). In order to evaluate the clinical value, we calculated the sensitivity (SE) and specificity (SP) using the results obtained by routine OROV rt-PCR as reference values. Based on these data, the SE and SP of OROV detection were, respectively, 100% [95% CI, 59.04 to 100] and 50% [95% CI, 1.26 to 98.74] for whole blood (with the routine rt-PCR Ct value range of 24.51–40.67). For serum, the SE and SP were 100% [95% CI, 47.82 to 100.00] and 100% [95% CI, 54.07 to 100.00], respectively (with the routine rtPCR Ct value range of 22.60–32.93). For urine, the SE and SP were both 75% [95% CI, 19.41 to 99.37]. Of note, we observed that the discordant results had Ct value ≥34 (by the routine rt-PCR). In particular, two whole blood samples (Ct 37.89 and 40.67) and one urine (Ct 39.13) sample were detected with <3 droplets. One urine specimen (Ct 38.50) tested negative even when repeated. Moreover, among the negative results, one whole blood sample and one urine sample were tested to be weakly positive (with <3 droplets) by ddPCR. Concerning *RPP30*, it was detected in all samples of the total cohort. For all the experiments, the negative and positive controls were tested and verified.

## 4. Discussion

Oropouche fever has clinical manifestations closely mimicking those of other arboviral infections as Dengue and Chikungunya viruses, this poses a significant challenge for diagnosis, necessitating laboratory confirmation. This scenario drives many One Health system challenges for vigilance and disease identification and control. As of today, no studies have reported the use of ddPCR for the detection of OROV. In order to support the monitoring programs, the purpose of this study was to provide preliminary data about the ddPCR using PAHO assay (designed and validated for Real-Time PCR) [12]. Overall, our results showed a strong correlation between TCID_50_/mL and number of RNA copies per µL, suggesting that the PAHO ddPCR assay can meet the need for a rapid and accurate determination of the viral titer, hence representing a good compromise for diagnostic or research laboratory. Overall, our LoD analyses detected 1 cp/µL, in line with Naveca et al.’s results [12] showing that PAHO Real-Time PCR assay had an LoD between 2 and 20 copies per reaction (Mean Ct values, 34.8–38.1) in serum. As a novel finding, here, we have shown the LoD measurements for urine and whole blood, suggesting the inclusion of these biological fluids, in addition to serum, for OROV investigation as we previously mentioned [9]. Of note, compared to the serum and whole blood data, the urine data were not completely equal in terms of quantification compared to the TCID_50_/mL, leading to further investigations to clarify whether the sample type may influence the detection rate as well as possibly find the optimal dilution to reduce potential reaction inhibitors [17].

Regarding the clinical samples, specificity and sensitivity suggest higher sensitivity of ddPCR compared to rt-PCR, but the estimated values may not be very precise because the CI intervals are wide. Of note, our analysis considered samples positive even if there were < 3 droplets and thus as detection data (positive/negative). On the other hand, for an accurate quantification, it is usually suggested to use a cut-off of at least three droplets as recommended by the manufacturer’s user guide [18]. Generally, for a weak positive sample, multiple well analysis and merge of quantification measurement could be indicated in order to achieve a more accurate analysis. Positive and negative controls are essential for clinical diagnosis [19], and they were tested and verified in our experiments, suggesting no false positivity in our cohort. Moreover, our work did not characterize viral load kinetics in OROV infection and, for a more accurate viral load quantification, it should be taken into account that viral loads can be expected to be relatively high in the diagnosis setting compared to the follow-up. The detection of OROV in the urine strongly suggests that OROV sheds into additional body fluids other than blood and CSF, as formerly reported [9]. To our knowledge, two previous studies have described the detection of OROV in the urine of infected patients [20,21] and, in light of our results, it is possible to recommend this specimen as a supplementary source for the detection of OROV.

Concerning the *RPP30,* it was generally measured to have a similar signal in all the biological matrices, highlighting a specific quantification measurement in the whole blood, serum, and urine. For the purpose of our study, we did not consider the use of additional reference genes for the different specimens analyzed. Indeed, we chose *RPP30* as an indicator for cellular content [22,23,24] and, in order to assess it, a simple duplex assay was performed in a unique reaction. For arthropod studies, further investigation should include other vector genomic indicators.

To conclude, with this work, we have provided for the first time the usage of ddPCR for the RNA detection and quantification of OROV. Our results should be considered preliminary for the investigation on three different biological matrices, including whole blood and urine in addition to serum, recommended for the OROV clinical laboratory investigation. Based on the small number of our available OROV-infected cohort, a larger number of samples and data from other laboratories could increase the clinical value of ddPCR method presented herein. In particular, the wide CI intervals can reflect estimated values of sensitivity and specificity that are not very precise due to the small size of samples, and thus the analysis should be extended to a larger cohort. We also suggest, for example, the important application of monitoring the viral load dynamic of OROV during follow-up. In addition to the laboratory diagnosis, ddPCR can help to shed light on the pathogenesis, filling in the knowledge gaps of this neglected disease, and to formulate the vector control programs.

## Figures and Tables

**Table 1:** Summarized results of ddPCR in the clinical samples detected using in house real time rtPCR. CI, confidence interval; SE, sensitivity; SP, specificity.

		In House rt-PCR (Reference)			
Specimen	ddPCR	Positive	Negative	SE [95% CI]	SP [95% CI]
Whole blood	Positive	7	1	100% [59.04 to 100]	50% [1.26 to 98.74]
	Negative	0	1		
Serum	Positive	5	0	100% [47.82 to 100.00]	100% [54.07 to 100.00]
	Negative	0	6		
Urine	Positive	3	1	75% [19.41 to 99.37]	75% [19.41 to 99.37]
	Negative	1	3		

## Data Availability

All data generated or analyzed during this study are included in this published article (and its supplementary information files).

## Supplementary Materials

The following supporting information can be downloaded at: https://www.mdpi.com/article/10.3390/v16091426/s1, Table S1: ddPCR LoD results in water; Table S2: ddPCR LoD results in biological matrix; Table S3: ddPCR results obtained in clinical positive samples.
